# Contemporary physiotherapy interventions for balance rehabilitation in children with Down syndrome: a systematic review of randomized controlled trials

**DOI:** 10.1007/s00431-026-07255-0

**Published:** 2026-07-27

**Authors:** Dimitra Kanopoulou, Alexandros Norikov, Zacharias Dimitriadis, Konstantinos Chandolias, Christina Vassou, Thomas Besios

**Affiliations:** 1 Department of Physiotherapy, Human Performance & Rehabilitation Laboratory, School of Health Sciences, University of Thessaly, 3Rd Km Old National Road Lamia-Athens, 35100 Lamia, Greece; 2 Department of Physiotherapy, School of Health Sciences, University of Thessaly, 3Rd Km Old National Road Lamia-Athens, 35100 Lamia, Greece

**Keywords:** Down syndrome, Physiotherapy, Balance, Pediatric rehabilitation, Postural control, Randomized controlled trials

## Abstract

Children with Down syndrome commonly present with impaired balance and postural control due to musculoskeletal, sensory, and neuromotor deficits, which may negatively affect functional mobility and participation in daily activities. Over the last decade, a variety of contemporary physiotherapy interventions have been investigated to improve balance in this population. The aim of this systematic review was to synthesize evidence from randomized controlled trials evaluating physiotherapy interventions targeting balance rehabilitation in children with Down syndrome. A systematic search was conducted in PubMed and Scopus for studies published between 2015 and 2025. Randomized controlled trials investigating physiotherapy-related interventions targeting balance in children and adolescents with Down syndrome were included. Two independent reviewers performed study selection and data extraction. Methodological quality was assessed using the Physiotherapy Evidence Database (PEDro) scale. A total of 14 randomized controlled trials involving 462 participants were included. The included interventions comprised isokinetic training, trampoline-based plyometric exercise, Pilates, dual-task exercise, hippotherapy, whole-body vibration, foot muscle strengthening, swimming programs, kinesiotaping, and vestibular stimulation. Improvements in balance-related outcomes were observed across a broad range of physiotherapy interventions, particularly when approaches were combined with conventional physiotherapy programs.

*Conclusion*: Contemporary physiotherapy interventions appear to be associated with improvements in balance-related outcomes in children with Down syndrome. The findings of this review support the integration of technology-assisted and task-oriented rehabilitation approaches into pediatric physiotherapy practice. However, the overall methodological quality of the included studies ranged from fair to high, and further large-scale, high-quality RCTs with long-term follow-up are required to strengthen the current evidence base.

**Supplementary Information:**

The online version contains supplementary material available at 10.1007/s00431-026-07255-0.

**Table:** 

**What is Known:** • *Children with Down syndrome commonly present with impaired balance and postural control, which negatively affect functional mobility and participation in daily activities.* • *A variety of physiotherapy interventions have been investigated to improve balance in this population, but the available evidence has not been comprehensively synthesized.*
**What is New:** • *This systematic review provides an up-to-date synthesis of randomized controlled trials evaluating contemporary physiotherapy interventions for balance rehabilitation in children with Down syndrome.* • *Exercise-based, technology-assisted, and task-oriented physiotherapy interventions appear to be associated with improvements in balance and related motor outcomes, although methodological heterogeneity and limited long-term follow-up warrant cautious interpretation.*

## Introduction

### Down syndrome

Down syndrome (DS) is one of the most common chromosomal and developmental disorders and represents the leading genetic cause of intellectual disability in children [1]. Children with DS frequently present with multisystem impairments affecting the musculoskeletal, neurological, cardiovascular, and sensory systems, which may negatively influence motor development and functional performance [2]. Common clinical characteristics include generalized hypotonia, ligamentous laxity, muscle weakness, delayed motor development, joint hypermobility, and impaired postural control, all of which contribute to balance and gait deficits [3, 4]. DS is primarily caused by trisomy of chromosome 21 resulting from chromosomal nondisjunction, while less common forms include translocation and mosaicism [1]. Due to the motor and postural impairments associated with the condition, physiotherapy interventions play an essential role in improving balance, mobility, functional independence, and participation in daily activities among children with DS [2].

### Balance

Balance and postural control are fundamental components of human movement and functional independence. Balance is maintained through the coordinated interaction of the musculoskeletal and nervous systems, allowing the body to respond effectively to both internal and external perturbations [2]. Adequate postural stability depends on the integration of sensory information derived from the visual, vestibular, and somatosensory systems, which is processed by the central nervous system to generate appropriate motor responses [3]. Efficient balance control enables individuals to maintain stable postures, perform voluntary movements, and adapt to environmental challenges during daily activities.

Children with Down syndrome commonly demonstrate impaired balance and postural control due to hypotonia, ligamentous laxity, muscle weakness, delayed motor development, and sensory integration deficits [4]. These impairments may negatively affect gait, mobility, participation in physical activities, and overall functional independence. Consequently, balance rehabilitation constitutes a major therapeutic goal in pediatric physiotherapy for children with DS.

Previous systematic reviews have investigated gait characteristics, postural control, and gross motor function in individuals with Down syndrome, highlighting the importance of physiotherapy for improving motor performance and functional abilities [2–4]. However, these reviews primarily focused on describing motor impairments or specific rehabilitation outcomes rather than synthesizing evidence from contemporary randomized controlled trials evaluating physiotherapy interventions specifically targeting balance rehabilitation.

Conventional physiotherapy for children with Down syndrome has traditionally focused on strengthening exercises, stretching, balance training, gait training, and functional motor activities. In recent years, however, a wide range of physiotherapy interventions have emerged, including technology-assisted rehabilitation, dual-task training, task-oriented exercise programs, and other innovative therapeutic approaches. Despite the growing body of evidence, there is still no clear consensus regarding which interventions are most appropriate for improving balance in this population. Therefore, the aim of this systematic review was to synthesize the available evidence from randomized controlled trials on contemporary physiotherapy interventions for balance rehabilitation in children with Down syndrome.

## Methodology

This systematic review was conducted according to the PRISMA 2020 (Preferred Reporting Items for Systematic Reviews and Meta-Analyses) guidelines and flowchart (Page et al. 2021).

### Eligibility criteria

To conduct this systematic review, certain eligibility criteria were established, and specific keywords were used.

Studies were considered eligible if they fulfilled the following inclusion criteria:

- Published exclusively in the English language,
- That were RCTs,
- Involving children and adolescents aged up to 20 years,
- In which all participants had a diagnosis of Down syndrome,
- In which the participants were able to understand and follow instructions,
- Investigating physiotherapy interventions targeting balance rehabilitation,
- That assessed balance using a valid and reliable outcome measure,
- Published between 2015 and 2025,
- Including any type of comparison group (e.g., conventional physiotherapy, usual care, sham intervention, or no intervention).

On the contrary, the exclusion criteria involved studies:

- That were nonrandomized studies, study protocols, conference abstracts, or review articles,
- Involving participants with severe comorbidities (e.g., neurological, musculoskeletal, or cardiovascular conditions) that could independently affect balance performance or participation in physiotherapy interventions,
- Studies including mixed clinical populations in which results for participants with Down syndrome could not be analyzed separately.

Only studies published in English were considered eligible because translation resources for studies published in other languages were not available.

Studies employing any eligible comparison condition were considered for inclusion, provided that all other eligibility criteria were met.

### Information sources, search strategies, and selection process

A systematic literature search was conducted on February 10, 2025, using the electronic databases PubMed and Scopus. The search was restricted to studies published between 2015 and 2025 in order to capture contemporary rehabilitation approaches currently applied in pediatric physiotherapy practice. The review protocol was registered in the International Prospective Register of Systematic Reviews (PROSPERO, registration number CRD420250655600).

The search strategy was developed using combinations of keywords and Medical Subject Headings (MeSH) related to Down syndrome, balance, physiotherapy, rehabilitation, exercise, and children. Boolean operators (“AND,” “OR”) were applied appropriately to combine search terms. The search was limited to randomized controlled trials published in English between 2015 and 2025.

The following keywords were used:

“Down syndrome,” “children,” “balance,” “physiotherapy,” “rehabilitation,” and “therapeutic exercise.”

Two independent reviewers screened the studies based on titles, abstracts, and full texts according to the predefined eligibility criteria. In cases of disagreement, a third reviewer was available to resolve discrepancies; however, consensus was achieved without requiring arbitration.

Additionally, the reference lists of the included studies were manually screened to identify potentially relevant articles.

Detailed search strategies for the selected databases are presented in Table 1.

**Table 1:** Complete electronic search strategy

Database	Search terms
PubMed	((“balance”[All Fields] OR “balanced”[All Fields] OR “balances”[All Fields] OR “balancing”[All Fields] OR (“child”[MeSH Terms] OR “child”[All Fields] OR “children”[All Fields] OR “child s”[All Fields] OR “children s”[All Fields] OR “childrens”[All Fields] OR “childs”[All Fields]) OR (“physical therapy modalities”[MeSH Terms] OR (“physical”[All20 Fields] AND “therapy”[All Fields] AND “modalities”[All Fields]) OR “physical therapy modalities”[All Fields] OR “physiotherapies”[All Fields] OR “physiotherapy”[All Fields]) OR (“exercise therapy”[MeSH Terms] OR (“exercise”[All Fields] AND “therapy”[All Fields]) OR “exercise therapy”[All Fields] OR (“therapeutic”[All Fields] AND “exercise”[All Fields]) OR “therapeutic exercise”[All Fields]) OR (“rehabilitant”[All Fields] OR “rehabilitants”[All Fields] OR “rehabilitate”[All Fields] OR “rehabilitated”[All Fields] OR “rehabilitates”[All Fields] OR “rehabilitating”[All Fields] OR “rehabilitation”[MeSH Terms] OR “rehabilitation”[All Fields] OR “rehabilitations”[All Fields] OR “rehabilitative”[All Fields] OR “rehabilitation”[MeSH Subheading] OR “rehabilitation s”[All Fields] OR “rehabilitational”[All Fields] OR “rehabilitator”[All Fields] OR “rehabilitators”[All Fields])) AND (“down syndrome”[MeSH Terms] OR (“down”[All Fields] AND “syndrome”[All Fields]) OR “down syndrome”[All Fields])) AND ((randomizedcontrolledtrial[Filter]) AND (2015:2025[pdat]))
Scopus	TITLE-ABS-KEY((“Down syndrome”) AND (“balance” OR “postural control”) AND (“children”) AND (“physiotherapy” OR “rehabilitation”))

Searches were conducted on February 2025

*MeSH* Medical Subject Headings

### Data collection and items extracted

Data were extracted from the studies independently by the two reviewers, without seeking additional information from the authors.

The extracted data included the following:

- Sample characteristics (age, sex, comorbidities)
- The type of intervention implemented (e.g., strengthening exercises, interventions incorporating assistive technology) and the corresponding parameters (e.g., number and duration of sessions, total intervention period)
- The balance assessment tool employed in each study.

### Methodological quality assessment of the trials

The methodological quality of the included studies was assessed using the Physiotherapy Evidence Database (PEDro) scale. The PEDro scale was selected because it is specifically designed to evaluate the methodological quality of randomized controlled trials in physiotherapy and rehabilitation research and has demonstrated good reliability and validity [5, 6] The PEDro scale consists of 11 criteria, 10 of which assess internal validity, while the first criterion relates to external validity and is not included in the final score. Each criterion met contributes 1 point to the total score, which ranges from 0 to 10. Studies scoring below 4 are considered to be low quality, scores of 4–5 indicate fair quality, scores of 6–8 indicate high quality, and scores of 9–10 are classified as excellent quality [7]. It must be noted that the assessment was conducted by the two reviewers, and in case of disagreement, any differences were resolved through discussion.

### Synthesis methods

Due to the substantial clinical and methodological heterogeneity across the included studies, a quantitative meta-analysis was not considered appropriate. Heterogeneity was observed with respect to intervention type, treatment duration and frequency, comparator conditions, participant characteristics, and balance outcome measures. Therefore, a narrative synthesis was performed.

The included studies were grouped according to the primary type of physiotherapy intervention (e.g., technology-assisted interventions, exercise-based interventions, and functional or multimodal rehabilitation approaches). Comparator groups, including conventional physiotherapy, usual care, sham intervention, and no-intervention control groups, were considered according to the original study design and interpreted within each individual study rather than being pooled across studies.

Because the included studies employed different validated instruments to assess balance and related functional outcomes (e.g., Pediatric Balance Scale, Biodex Balance System, Timed Up and Go Test, Bruininks–Oseretsky Test of Motor Proficiency, Functional Reach Test, and WeeFIM), quantitative comparison of outcome measures was not feasible. Instead, the results were synthesized descriptively by describing the findings of individual studies according to intervention type and outcome measures while considering the methodological quality of the included studies.

## Results

### Study selection

The electronic database search identified 117 records, including 59 records from PubMed and 58 records from Scopus. After removal of 25 duplicate records, 92 studies remained for title and abstract screening. Following the screening process, 28 full-text articles were assessed for eligibility.

**Fig. 1:**
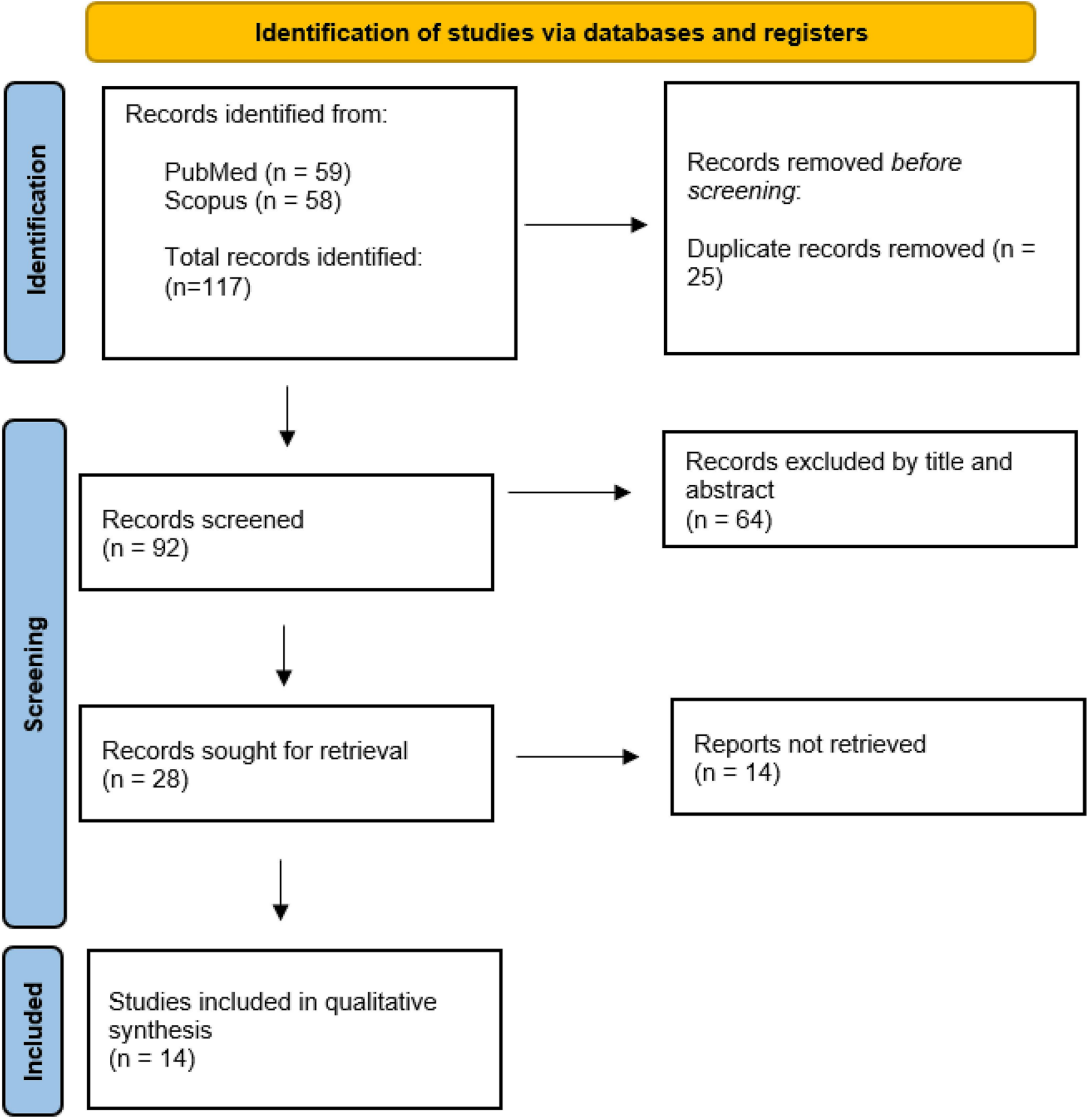
PRISMA 2020 flow diagram of the study selection process

Of these, 14 randomized controlled trials met the inclusion criteria and were included in the qualitative synthesis. Fourteen full-text articles were excluded following eligibility assessment because they did not meet the predefined inclusion criteria. The study selection process is illustrated in Fig. 1.

### Study characteristics

Fourteen randomized controlled trials (RCTs) were included in this systematic review. The studies were conducted across multiple countries, including Egypt, Turkey, Pakistan, Poland, India, Iran, and Tunisia. A total of 462 participants were included, with both sexes represented across the included studies. The duration of the interventions ranged from 4 to 33 weeks.

The assessment instruments used across the included studies comprised the Pediatric Balance Scale, Bruininks–Oseretsky Test of Motor Proficiency, Biodex Stability/Balance System, Timed Up and Go Test, Functional Reach Test, Gross Motor Function Measure-88, Functional Independence Measure for Children (WeeFIM), Four Square Step Test, Test of Gross Motor Development-2, Eurofit Test Battery, Single Leg Stance Test, Tandem Stance Test, 30-Second Sit-to-Stand Test, and the Modified Clinical Test of Sensory Interaction on Balance.

The included interventions comprised core stability exercise, dual-task exercise, foot muscle strengthening exercise, kinesiotaping, isokinetic training, whole-body vibration, mechanical vestibular stimulation, traditional Indian dance, trampoline-based plyometric exercise, swimming programs, ACSM-based physical exercise programs, hippotherapy, Pilates exercise, and vestibular and balance-oriented rehabilitation approaches.

Most studies (10/14) compared the investigated intervention with an active control condition, typically conventional physiotherapy. Three studies used a no-intervention or usual-care control group, while one study employed a sham intervention. No studies compared the intervention with education-only or wait-list control conditions.

The characteristics of the included studies are presented in detail in Table 2.

**Table 2:** Characteristics of the studies

Study	Sample	Assessment instrument	Method	Results
Eid [8]	30 children with Down syndrome Control group: *n* = 15, mean age 9.26 ± 0.79 years (9 males, 6 females) Intervention group: *n* = 15, mean age 8.93 ± 0.70 years (8 males, 7 females)	Biodex Stability System	Control group: conventional physiotherapy program including stretching, isometric strengthening, and balance exercises for 60 min, 3 sessions/week for 6 months Experimental group: the same physiotherapy program combined with an additional 5–10 min of whole-body vibration (WBV) training, 3 sessions/week for 6 months	Significantly greater improvements were observed in the whole-body vibration group compared with the conventional physiotherapy group
Eid et al. [9]	31 children with Down syndrome Control group: *n* = 16, mean age 10.05 ± 0.68 years (9 males, 7 females) Intervention group: *n* = 15, mean age 10.26 ± 0.79 years (8 males, 7 females)	Biodex System 3	Control group: conventional physiotherapy program for 60 min, 3 sessions/week for 12 weeks Experimental group: conventional physiotherapy for 45 min combined with 15 min of isokinetic training, 3 sessions/week for 12 weeks	Both groups demonstrated statistically significant improvements in postural stability indices following the intervention period. The authors reported significantly greater improvements in the isokinetic training group compared with the conventional physiotherapy group
Alsakhawi and Elshafey [10]	45 children with Down syndrome (mean age 4.59 ± 0.53 years) Group A: *n* = 15 Group B: *n* = 15 Group C: *n* = 15	Berg Balance Scale, Biodex Balance System	Group A: conventional physiotherapy program for 60 min, including standing and weight-shifting exercises Group B: 30 min of the conventional program combined with treadmill training, stretching, and cool-down exercises, applied 3 sessions/week for 8 weeks Group C: 30 min of the conventional physiotherapy program combined with Jeffrey core stability exercises, applied 3 sessions/week for 8 weeks	All three groups demonstrated statistically significant improvements in balance outcomes following the intervention period. The authors reported significantly greater improvements in groups B and C compared with group A, whereas no statistically significant differences were observed between groups B and C
Naczk [11]	22 adolescents with Down syndrome Control group: *n* = 11, mean age 14.4 ± 1.97 years (7 males, 4 females) Intervention group: *n* = 11, mean age 14.9 ± 2.35 years (7 males, 4 females)	Eurofit Test Battery (including Flamingo Balance Test)	Control group: participants maintained their usual daily activities throughout the study period Experimental group: progressive water-based exercise program conducted 3 sessions/week for 33 weeks	Both groups demonstrated similar balance performance following the intervention period, with no statistically significant between-group differences reported
Azab et al. [12]	31 children with Down syndrome Control group: *n* = 15, mean age 8.60 ± 0.98 years (11 males, 4 females) Intervention group: *n* = 16, mean age 9.19 ± 0.75 years (9 males, 7 females)	Biodex Balance System	Control group: standard physiotherapy program including strengthening exercises, balance training, and walking on different surfaces for 45 min, applied 2 sessions/week for 12 weeks Experimental group: the same physiotherapy program combined with an additional 15 min of trampoline-based plyometric exercises, applied 2 sessions/week for 12 weeks	Both groups demonstrated statistically significant improvements in balance performance following the intervention period. The authors reported significantly greater improvements in the trampoline-based plyometric exercise group compared with the conventional physiotherapy group
Nahla et al. [13]	30 children with Down syndrome Group A: *n* = 15, mean age 8.26 ± 1.16 years (11 males, 4 females) Group B: *n* = 15, mean age 8.33 ± 1.11 years (10 males, 5 females)	Biodex Balance System	Group A: conventional physiotherapy program including core strengthening, facilitation of balance reactions, postural transitions, and balance exercises for 60 min, applied 3 sessions/week for 3 months Group B: the same conventional physiotherapy program combined with 15 min of mechanical vestibular stimulation using a three-dimensional motion system, applied 3 sessions/week for 3 months	Both groups demonstrated statistically significant improvements in balance performance following the intervention period. Significantly greater improvements were reported in the group receiving mechanical vestibular stimulation compared with the conventional physiotherapy group
Raghupathy et al. [14]	36 children with Down syndrome Control group: *n* = 18, mean age 8.4 ± 1.3 years (10 males, 8 females) Intervention group: *n* = 18, mean age 7.8 ± 1.3 years (11 males, 7 females)	Four Square Step Test, Pediatric Balance Scale, Test of Gross Motor Development-2 (TGMD-2)	Control group: neuromuscular training program including strengthening, balance, and coordination exercises with warm-up and cool-down activities, applied 3 sessions/week for 6 weeks Intervention group: program consisting of traditional Indian dance sequences, applied 3 sessions/week for 6 weeks	Both intervention groups demonstrated improvements in balance and motor performance following the intervention period. The Indian dance group demonstrated significantly greater improvements in locomotor performance and dynamic balance (FSST), whereas no statistically significant between-group differences were reported for the Pediatric Balance Scale
Büyükçelik et al. [15]	27 children with Down syndrome (18 males, 9 females) Control group: *n* = 14, mean age 11.86 ± 4.09 years Intervention group: *n* = 13, mean age 12.08 ± 2.56 years	Functional Independence Measure for Children (WeeFIM), Pediatric Balance Scale, Timed Up-and-Go Test, Single Leg Stance Test, Tandem Stance Test, 30-Second Sit-to-Stand Test	Control group: no structured exercise intervention was implemented during the study period Intervention group: dual-task exercise program consisting of motor activities combined with cognitive tasks, including walking, squats, and single-leg stance exercises. Sessions lasted 30 min and were applied 2 sessions/week for 8 weeks	The dual-task intervention group demonstrated statistically significant improvements in balance, functional performance, and dual-task-related outcomes. In the control group, statistically significant improvement was observed only for the Pediatric Balance Scale, while no significant changes were reported for the remaining outcome measures
Kashi et al. [16]	36 adolescents with Down syndrome Control group: school activity program, *n* = 18, mean age 13.22 ± 1.93 years Intervention group: SSRI protocol, *n* = 18, mean age 12.55 ± 2.77 years	Bruininks–Oseretsky Test of Motor Proficiency (BOT-2)	Control group: participants followed the standard school activity program Intervention group: multimodal ACSM-based exercise package. Sessions lasted 1 h and were applied 2–3 sessions/week for 12 weeks	The intervention group demonstrated statistically significant improvements in motor proficiency, balance, coordination, muscular strength, flexibility, and functional motor performance compared with the school activity program
Kaya et al. [17]	34 children with Down syndrome Control group: mean age 8.18 ± 2.74 years Intervention group: mean age 10.12 ± 3.30 years	Pediatric Balance Scale, Timed Up and Go Test, Functional Independence Measure for Children (WeeFIM)	Both groups: balance exercise program including single-leg stance, tandem walking, and squatting exercises for 30 min, applied 3 sessions/week for 6 weeks Intervention group: additional hippotherapy sessions consisting of warm-up activities and horse-riding exercises targeting sensory integration, coordination, balance, and strength. Sessions lasted 30 min and were applied over a 6-week period	Both groups demonstrated statistically significant improvements in balance and functional mobility. Statistically significant improvements in functional independence (WeeFIM) were observed only in the hippotherapy group
Adeeb et al. [18]	47 children with Down syndrome Control group: *n* = 24, mean age 10.63 ± 2.74 years (15 males, 9 females) Intervention group: *n* = 23, mean age 10.0 ± 3.10 years (7 males, 16 females)	Gross Motor Function Measure-88 (GMFM-88), Pediatric Balance Scale (PBS)	Control group: individualized insoles combined with single-leg balance exercises and stretching activities for warm-up and cool-down, applied 3 sessions/week for 12 weeks Intervention group: foot muscle strengthening exercises combined with single-leg balance exercises and stretching activities. Sessions lasted 40 min and were applied 3 sessions/week for 12 weeks	Both groups demonstrated statistically significant improvements in gross motor function and balance following the intervention period. At follow-up, statistically significant improvements in both GMFM-88 and PBS were maintained in the foot muscle strengthening group, whereas only PBS remained significantly improved in the control group
Al-Nemr and Reffat [19]	40 children with Down syndrome Control group: *n* = 20, mean age 9.16 ± 0.48 years (6 males, 14 females) Intervention group: *n* = 20, mean age 8.86 ± 0.58 years (8 males, 12 females)	Biodex Balance System, Bruininks–Oseretsky Test of Motor Proficiency-2 (BOT-2)	Control group: physiotherapy program including flexibility, strengthening, balance, and walking exercises for 90 min, applied 3 sessions/week for 12 weeks Intervention group: conventional physiotherapy for 45 min combined with 45 min of Pilates exercises, including warm-up and cool-down activities, applied 3 sessions/week for 12 weeks	Both groups demonstrated statistically significant improvements in balance and motor proficiency outcomes. Significantly greater improvements were observed in the Pilates-based intervention group compared with the conventional physiotherapy group
Atalan Efkere and Tarsuslu [20]	24 children and adolescents with Down syndrome Sham group: *n* = 12, aged 9–18 years (4 males, 8 females) Kinesiotaping group: *n* = 12, aged 11–18 years (5 males, 7 females)	Functional Reach Test, FAST Timed Up-and-Go Test, Modified Clinical Test of Sensory Interaction on Balance (MCTSIB)	Sham group: application of tape without therapeutic placement Kinesiotaping group: application of kinesiotape to the plantar fascia aiming to provide sensory stimulation and mechanical support	Both the kinesiotaping and sham groups demonstrated statistically significant improvements in the Functional Reach Test and FAST-Timed Up-and-Go Test, whereas no statistically significant changes were observed in the Modified Clinical Test of Sensory Interaction on Balance. No statistically significant between-group differences were reported for any of the assessed outcomes
Triki et al. [21]	29 adolescents with Down syndrome Control group: *n* = 9, aged 14–16 years Single-task group: *n* = 10, aged 12–17 years Dual-task group: *n* = 10, aged 14–17 years	Static Stabilometric Platform	Control group: participants maintained their usual daily activities without structured intervention Single-task group: motor exercise program consisting of squats, jumps, walking exercises, and additional motor activities. Sessions lasted 45–60 min, including warm-up and cool-down periods, and were applied 3 sessions/week for 8 weeks Dual-task group: the same motor exercise program combined with cognitive tasks, applied 3 sessions/week for 8 weeks	The dual-task intervention group demonstrated statistically significant improvements in postural performance under both single-task and dual-task conditions. The single-task intervention group demonstrated statistically significant improvement only under the soft-surface condition, whereas no statistically significant changes were observed in the control group

### Quality of studies

The methodological quality of the studies included in this systematic review was assessed using the Physiotherapy Evidence Database (PEDro) scale. Overall, the methodological quality of the included studies ranged from low to high. One study was classified as low quality, five studies demonstrated fair methodological quality, and eight studies were considered high quality. The mean PEDro score of the included studies was 6.0, indicating an overall fair methodological quality.

Detailed PEDro scores for each included study are presented in Table 3.

**Table 3:** Scoring of the studies on the PEDro scale

Study	1^*^	2	3	4	5	6	7	8	9	10	11	Scoring	Quality
Eid [8]	1	1	1	1	0	0	1	1	1	1	1	8/10	High
Eid et al. [9]	1	1	1	1	0	0	1	1	1	1	1	8/10	High
Alsakhawi and Elshafey [10]	1	1	0	1	0	0	0	0	0	1	1	4/10	Fair
Naczk et al. [11]	1	1	0	1	0	0	0	1	0	1	1	5/10	Fair
Azab et al. [12]	1	1	1	1	0	0	1	1	0	1	1	7/10	High
Nahla et al. [13]	1	1	0	1	0	0	0	0	0	1	1	4/10	Fair
Raghupathy et al. [14]	1	1	1	1	0	0	0	1	1	1	1	7/10	High
Büyükçelik et al. [15]	1	1	0	1	0	0	1	1	0	1	1	6/10	High
Kashi et al. [16]	1	1	0	0	0	0	0	1	0	0	1	3/10	Low
Kaya et al. [17]	1	1	0	1	0	0	0	1	0	1	1	5/10	Fair
Adeeb et al. [18]	1	1	1	1	0	0	0	1	0	1	1	6/10	High
Al-Nemr and Reffat [19]	1	1	1	1	0	0	1	0	0	1	1	6/10	High
Efkere and Tarsuslu (2024)	1	1	0	1	0	0	1	1	1	1	1	7/10	High
Triki et al. [21]	1	1	1	1	0	0	1	1	1	1	1	8/10	High

^*^Item 1 (eligibility criteria) relates to external validity and is not included in the total PEDro score

### Effectiveness of the interventions

Overall, balance-related outcomes appeared to improve across a wide range of physiotherapy interventions included in this review. The interventions investigated varied considerably regarding therapeutic approach, duration, intensity, and outcome measures.

Technology-assisted interventions were investigated in several studies and appeared to be associated with improvements in postural stability and balance-related outcomes. Whole-body vibration training [8], mechanical vestibular stimulation [13], and isokinetic training [9] appeared to be associated with improvements in postural stability and balance-related outcomes following the intervention period.

Exercise-based interventions were also associated with improvements in balance-related outcomes. Core stability and treadmill-based approaches [10], trampoline-based plyometric exercise [12], dual-task exercise interventions [15, 21], Pilates-based exercise programs [19], an ACSM-based multimodal exercise program incorporating aerobic, strengthening, balance, coordination, and functional exercises [16], and foot muscle strengthening exercises [18] were all associated with improvements in balance-related outcomes. Several of these interventions also demonstrated beneficial effects on motor performance, functional mobility, and gross motor function.

Interventions targeting motor coordination and functional mobility also demonstrated encouraging findings. Traditional Indian dance [14] and hippotherapy [17] appeared to be associated with improvements in motor proficiency, mobility, and balance-related measures.

In contrast, more limited changes in balance performance were reported in studies investigating swimming-based exercise programs [11] and kinesiotaping interventions [20].

## Discussion

The present systematic review aimed to investigate contemporary physiotherapy interventions targeting balance rehabilitation in children with Down syndrome. Overall, the findings of the included randomized controlled trials suggest that a wide range of physiotherapy interventions may be associated with improvements in balance-related outcomes. Although the included studies generally reported encouraging findings, considerable clinical and methodological heterogeneity was observed with respect to intervention protocols, treatment duration, assessment tools, and reported outcomes.

Exercise-based rehabilitation approaches constituted the most frequently investigated interventions. Core stability and balance-oriented exercise programs appeared to be commonly associated with improvements in postural control and functional balance outcomes [10]. Similarly, interventions incorporating trampoline-based plyometric exercise (Azab et al. 12, Pilates-based exercise [19], and dual-task training protocols [15, 21] were associated with improvements in balance, functional mobility, and motor performance. These findings may indicate a potential role of task-oriented and neuromuscular exercise approaches in addressing the postural and motor deficits commonly observed in children with Down syndrome.

Technology-assisted rehabilitation interventions were investigated in several studies and were associated with improvements in balance-related outcomes. Whole-body vibration training [8], isokinetic training [9], and mechanical vestibular stimulation [13] were associated with improvements in balance-related measures following the intervention period. Potential mechanisms underlying these findings may involve alterations in sensory input, neuromuscular activation, and postural responses, which could contribute to postural stability and motor control.

In addition, interventions focusing on functional mobility and motor coordination were associated with improvements in balance-related and functional outcomes. Traditional Indian dance [14], hippotherapy [17], and multimodal exercise programs based on ACSM principles [16] were associated with improvements in balance, motor proficiency, and functional mobility outcomes. Foot muscle strengthening exercises also demonstrated favorable effects regarding balance and gross motor function [18]. These findings support the growing interest in functional, engaging, and participation-oriented rehabilitation approaches for children with Down syndrome.

In contrast, more limited changes in balance performance were reported in studies investigating swimming-based exercise programs [11] and kinesiotaping interventions [20]. However, interpretation of these outcomes should be performed cautiously due to differences in intervention protocols, outcome measures, and study characteristics.

The methodological quality of the included studies ranged from low to high according to the PEDro scale. Nevertheless, methodological heterogeneity, relatively small sample sizes, and limited long-term follow-up should be considered when interpreting the findings.

This systematic review also presents several limitations. First, considerable clinical and methodological heterogeneity was observed across the included studies regarding intervention protocols, treatment duration, comparator conditions, and outcome measures, precluding quantitative synthesis of the findings. Furthermore, most studies included relatively small sample sizes and were restricted to children without severe comorbidities, limiting the generalizability of the findings. In addition, the majority of the included studies evaluated only short-term intervention effects, with limited or no long-term follow-up, making it difficult to determine the sustainability of the reported improvements. Finally, methodological quality was assessed using the PEDro scale rather than the Cochrane Risk of Bias 2.0 tool, which may have provided a more comprehensive domain-based assessment of risk of bias.

Future research should prioritize large-scale, high-quality randomized controlled trials with standardized intervention protocols and long-term follow-up assessment. Greater methodological consistency regarding outcome measures and intervention reporting would facilitate comparison across studies and strengthen the current evidence base regarding physiotherapy interventions for balance rehabilitation in children with Down syndrome.

## Conclusion

The findings of this systematic review support the role for physiotherapy interventions in balance rehabilitation and functional performance in children with Down syndrome. Several rehabilitation approaches demonstrated encouraging findings regarding balance and functional outcomes.

Technology-assisted interventions were frequently associated with improvements in balance-related outcomes. At the same time, interventions such as dual-task exercise and traditional Indian dance emphasized the importance of functional, engaging, and participation-oriented therapeutic approaches that may enhance motivation and active involvement during rehabilitation.

Despite the generally positive findings, the heterogeneity observed across intervention protocols, treatment duration, and outcome measures highlights the need for cautious interpretation of the current evidence. Further high-quality randomized controlled trials with larger sample sizes, standardized methodologies, and long-term follow-up assessments are required to strengthen the evidence base and support the development of optimized physiotherapy protocols for children with Down syndrome.

## Supplementary Information

Below is the link to the electronic supplementary material.

ESM 1 (DOCX 12.6 KB)

ESM 2 (DOCX 16.6 KB)

ESM 3 (DOCX 14.3 KB)

## Data Availability

No datasets were generated or analysed during the current study.
