# Supplementary material for: Contemporary physiotherapy interventions for balance rehabilitation in children with Down syndrome: a systematic review of randomized controlled trials
Source: Eur J Pediatr. 2026 Jul 27;185(8):615. doi: 10.1007/s00431-026-07255-0 (PMC13408123; doi:10.1007/s00431-026-07255-0)
Supplement: Supplementary file 1 — (DOCX 12.6 KB) [file 431_2026_7255_MOESM1_ESM.docx]

Detailed Search Strategy

**PubMed**

(("balance"[All Fields] OR "balanced"[All Fields] OR "balances"[All Fields] OR "balancing"[All Fields] OR ("child"[MeSH Terms] OR "child"[All Fields] OR "children"[All Fields] OR "child s"[All Fields] OR "children s"[All Fields] OR "childrens"[All Fields] OR "childs"[All Fields]) OR ("physical therapy modalities"[MeSH Terms] OR ("physical"[All20 Fields] AND "therapy"[All Fields] AND "modalities"[All Fields]) OR "physical therapy modalities"[All Fields] OR "physiotherapies"[All Fields] OR "physiotherapy"[All Fields]) OR ("exercise therapy"[MeSH Terms] OR ("exercise"[All Fields] AND "therapy"[All Fields]) OR "exercise therapy"[All Fields] OR ("therapeutic"[All Fields] AND "exercise"[All Fields]) OR "therapeutic exercise"[All Fields]) OR ("rehabilitant"[All Fields] OR "rehabilitants"[All Fields] OR "rehabilitate"[All Fields] OR "rehabilitated"[All Fields] OR "rehabilitates"[All Fields] OR "rehabilitating"[All Fields] OR "rehabilitation"[MeSH Terms] OR "rehabilitation"[All Fields] OR "rehabilitations"[All Fields] OR "rehabilitative"[All Fields] OR "rehabilitation"[MeSH Subheading] OR "rehabilitation s"[All Fields] OR "rehabilitational"[All Fields] OR "rehabilitator"[All Fields] OR "rehabilitators"[All Fields])) AND ("down syndrome"[MeSH Terms] OR ("down"[All Fields] AND "syndrome"[All Fields]) OR "down syndrome"[All Fields])) AND ((randomizedcontrolledtrial[Filter]) AND (2015:2025[pdat]))

**Scopus**

("Down syndrome")

AND ("balance" OR "postural control")

AND ("children")

AND ("physiotherapy" OR "rehabilitation")

Filters: English, 2015–2025
