# Supplementary material for: Contemporary physiotherapy interventions for balance rehabilitation in children with Down syndrome: a systematic review of randomized controlled trials
Source: Eur J Pediatr. 2026 Jul 27;185(8):615. doi: 10.1007/s00431-026-07255-0 (PMC13408123; doi:10.1007/s00431-026-07255-0)
Supplement: Supplementary file 2 — (DOCX 16.6 KB) [file 431_2026_7255_MOESM2_ESM.docx]

Supplementary PEDro Scale

| Study | 1* | 2 | 3 | 4 | 5 | 6 | 7 | 8 | 9 | 10 | 11 | Scoring | Quality |
| --- | --- | --- | --- | --- | --- | --- | --- | --- | --- | --- | --- | --- | --- |
| Eid (2015) | 1 | 1 | 1 | 1 | 0 | 0 | 1 | 1 | 1 | 1 | 1 | 8 / 10 | High |
| Eid et al (2017) | 1 | 1 | 1 | 1 | 0 | 0 | 1 | 1 | 1 | 1 | 1 | 8 / 10 | High |
| Alsakhawi & Elshafey (2019) | 1 | 1 | 0 | 1 | 0 | 0 | 0 | 0 | 0 | 1 | 1 | 4 / 10 | Fair |
| Naczk et al (2021) | 1 | 1 | 0 | 1 | 0 | 0 | 0 | 1 | 0 | 1 | 1 | 5 / 10 | Fair |
| Azab et al (2022) | 1 | 1 | 1 | 1 | 0 | 0 | 1 | 1 | 0 | 1 | 1 | 7 / 10 | High |
| Nahla et al (2022) | 1 | 1 | 0 | 1 | 0 | 0 | 0 | 0 | 0 | 1 | 1 | 4 / 10 | Fair |
| Raghupathy et al (2022) | 1 | 1 | 1 | 1 | 0 | 0 | 0 | 1 | 1 | 1 | 1 | 7 / 10 | High |
| Büyükçelik et al (2023) | 1 | 1 | 0 | 1 | 0 | 0 | 1 | 1 | 0 | 1 | 1 | 6 / 10 | High |
| Kashi et al (2023) | 1 | 1 | 0 | 0 | 0 | 0 | 0 | 1 | 0 | 0 | 1 | 3 / 10 | Low |
| Kaya et al (2023) | 1 | 1 | 0 | 1 | 0 | 0 | 0 | 1 | 0 | 1 | 1 | 5 / 10 | Fair |
| Adeeb et al (2024) | 1 | 1 | 1 | 1 | 0 | 0 | 0 | 1 | 0 | 1 | 1 | 6 / 10 | High |
| Al-Nemr & Reffat (2024) | 1 | 1 | 1 | 1 | 0 | 0 | 1 | 0 | 0 | 1 | 1 | 6 / 10 | High |
| Efkere & Tarsuslu (2024) | 1 | 1 | 0 | 1 | 0 | 0 | 1 | 1 | 1 | 1 | 1 | 7 / 10 | High |
| Triki et al (2024) | 1 | 1 | 1 | 1 | 0 | 0 | 1 | 1 | 1 | 1 | 1 | 8 / 10 | High |
