# Supplementary material for: Contemporary physiotherapy interventions for balance rehabilitation in children with Down syndrome: a systematic review of randomized controlled trials
Source: Eur J Pediatr. 2026 Jul 27;185(8):615. doi: 10.1007/s00431-026-07255-0 (PMC13408123; doi:10.1007/s00431-026-07255-0)
Supplement: Supplementary file 3 — (DOCX 14.3 KB) [file 431_2026_7255_MOESM3_ESM.docx]

Supplementary Table S3. Quantitative summary of the primary findings of the included studies.

| **Study** | **Primary balance outcome(s)** | **Between-group findings** | **Statistical significance** |
| --- | --- | --- | --- |
| Eid (2015) | Biodex Stability System | WBV + conventional physiotherapy produced greater improvement than conventional physiotherapy alone | p < 0.05 |
| Eid et al. (2017) | Biodex System 3 | Isokinetic training + conventional physiotherapy demonstrated greater improvements than conventional physiotherapy | p < 0.05 |
| Alsakhawi & Elshafey (2019) | Berg Balance Scale, Biodex Balance System | Treadmill and Jeffrey exercise groups demonstrated greater improvements than conventional physiotherapy; no difference between intervention groups | p < 0.05 |
| Naczk et al. (2021) | Flamingo Balance Test | No significant between-group difference | p > 0.05 |
| Azab et al. (2022) | Biodex Balance System | Trampoline-based plyometric exercise resulted in greater improvements than conventional physiotherapy alone | p < 0.05 |
| Nahla et al. (2022) | Biodex Balance System | Mechanical vestibular stimulation demonstrated greater improvements than conventional physiotherapy | p < 0.05 |
| Raghupathy et al. (2022) | PBS, FSST, TGMD-2 | Both interventions improved outcomes; no superiority clearly established | Significant within-group improvements reported |
| Büyükçelik et al. (2023) | PBS, TUG, WeeFIM | Dual-task exercise improved balance and functional performance compared with no intervention | p < 0.05 |
| Kashi et al. (2023) | BOT-2 | ACSM-based exercise package improved motor proficiency and balance compared with school activities | p < 0.05 |
| Kaya et al. (2023) | PBS, TUG, WeeFIM | Hippotherapy improved functional performance beyond conventional balance exercises | p < 0.05 |
| Adeeb et al. (2024) | PBS, GMFM-88 | Foot muscle strengthening showed greater improvements at follow-up than control intervention | p < 0.05 |
| Al-Nemr & Reffat (2024) | Biodex, BOT-2 | Pilates plus physiotherapy demonstrated greater improvements than physiotherapy alone | p < 0.05 |
| Atalan Efkere & Tarsuslu (2024) | FRT, FAST-TUG, MCTSIB | No significant between-group differences | p > 0.05 |
| Triki et al. (2024) | Static Stabilometric Platform | Dual-task training produced greater improvements than single-task training and control | p < 0.05 |
